# PS-14 - 2023-2025 HEPATITIS A OUTBREAK ACROSS EU AND UK ASSOCIATED WITH FRESH BERRIES WITH POTENTIAL ONGOING SEASONAL RISK: A RETROSPECTIVE CASE-CASE STUDY ACROSS THE FOUR NATIONS

**DOI:** 10.1093/pubmed/fdag046.061

**Published:** 2026-07-09

**Authors:** Mr Sawyer Patterson, Jin-Min Yuan, David Leeman, Koye Balogun, Tatiana Garcia Vilaplana, Neville Verlander, Siew Lin Ngui, Andre Charlett, Andrew Nelson, Alison Smith-Palmer, Caitlin McCarthy, Samantha Shepherd, Rory Gundson, Danielle McMichael, Patrick McAleavey, Miranda Mindlin

**Affiliations:** UK Health Security Agency; UK Health Security Agency; UK Health Security Agency; UK Health Security Agency; UK Health Security Agency; UK Health Security Agency; UK Health Security Agency; UK Health Security Agency; Public Health Wales; Public Health Scotland; Public Health Scotland; NHS Greater Glasgow and Clyde; NHS Greater Glasgow and Clyde; Public Health Agency Northern Ireland; Public Health Agency Northern Ireland; UK Health Security Agency

## Abstract

**Background:**

In 2025, a cluster of genetically-linked non-travel hepatitis A cases was identified in the UK and EU, phylogenetically clustering with historic hepatitis A sequences from travellers returning from Morocco. Food histories were routinely collected from the non-travel hepatitis A cases. An incident management team (IMT) was established, convening the public health agencies of the five nations and Food Standards Agency.

**Objectives:**

To investigate the causative exposure using a case-case study which will inform future actions and control measures.

**Methods:**

The IMT instigated a non-matched case-case study to identify the source of infection. Study cases had hepatitis A virus sequences within one nucleotide of the Moroccan-like VRD25_HAV016 sequence. Comparison case’s sequences were outside this genetic cluster. All were primary/co-primary non-travel hepatitis A cases and UK residents, with food histories and sample dates between 26th November 2024-3rd June 2025 (the outbreak peak). Firth’s logistic regression was used to test the association between being a study case and food exposures reported by ≥60% of study cases. UK food import data was utilised to explore importation of non-UK-grown berries as a possible source.

**Results:**

86 cases within the VRD25_HAV016 cluster were reported from 2023-2025: 61 were hospitalised, and 47 included in this study. Fresh berries (consumed by 85% of study cases) were significantly associated with being a study case (aOR 4.16, 95% CI: 1.52-13.20, p=0.005) after adjustment. Morocco was a major source of berries imported to the UK during the winter.

**Conclusions:**

Fresh berries are the likely source of this outbreak. Food import and phylogenetic data suggest contamination occurred abroad, reinforcing the importance of effective food safety measures from farm to fork to prevent further outbreaks. Effective multinational collaboration on data collection, sharing and analysis was essential for the response. Streamlining data-sharing and data collection may accelerate public health action across all five nations in future foodborne outbreaks.

